# Operative versus non-operative treatment for 2-part proximal humerus fracture: A multicenter randomized controlled trial

**DOI:** 10.1371/journal.pmed.1002855

**Published:** 2019-07-18

**Authors:** Antti P. Launonen, Bakir O. Sumrein, Aleksi Reito, Vesa Lepola, Juha Paloneva, Kenneth B. Jonsson, Olof Wolf, Peter Ström, Hans E. Berg, Li Felländer-Tsai, Karl-Åke Jansson, Daniel Fell, Inger Mechlenburg, Kaj Døssing, Helle Østergaard, Aare Märtson, Minna K. Laitinen, Ville M. Mattila, as the NITEP group

**Affiliations:** 1 Faculty of Medicine and Health Technology, University of Tampere and Tampere University Hospital, Tampere, Finland; 2 Department of Orthopaedics, Central Finland Central Hospital, Jyväskylä, Finland; 3 Department of Orthopaedics, Institute of Surgical Sciences, Uppsala University Hospital, Uppsala, Sweden; 4 Division of Orthopedics and Biotechnology, Department of Clinical Science, Intervention and Technology, Karolinska Institutet, Stockholm, Sweden; 5 Department of Orthopedics, Karolinska University Hospital, Huddinge, Sweden; 6 Department of Orthopaedic Surgery, Aarhus University Hospital, Aarhus, Denmark; 7 Department of Clinical Medicine, Aarhus University, Aarhus, Denmark; 8 Orthopedics Department, Viborg Regional Hospital, Viborg, Denmark; 9 Department of Traumatology and Orthopedics, Tartu University Hospital and Tartu University, Tartu, Estonia; Teesside University, UNITED KINGDOM

## Abstract

**Background:**

Although increasingly used, the benefit of surgical treatment of displaced 2-part proximal humerus fractures has not been proven. This trial evaluates the clinical effectiveness of surgery with locking plate compared with non-operative treatment for these fractures.

**Methods and findings:**

The NITEP group conducted a superiority, assessor-blinded, multicenter randomized trial in 6 hospitals in Finland, Estonia, Sweden, and Denmark. Eighty-eight patients aged 60 years or older with displaced (more than 1 cm or 45 degrees) 2-part surgical or anatomical neck proximal humerus fracture were randomly assigned in a 1:1 ratio to undergo either operative treatment with a locking plate or non-operative treatment. The mean age of patients was 72 years in the non-operative group and 73 years in the operative group, with a female sex distribution of 95% and 87%, respectively. Patients were recruited between February 2011 and April 2016. The primary outcome measure was Disabilities of Arm, Shoulder, and Hand (DASH) score at 2-year follow-up. Secondary outcomes included Constant–Murley score, the visual analogue scale for pain, the quality of life questionnaire 15D, EuroQol Group’s 5-dimension self-reported questionnaire EQ-5D, the Oxford Shoulder Score, and complications. The mean DASH score (0 best, 100 worst) at 2 years was 18.5 points for the operative treatment group and 17.4 points for the non-operative group (mean difference 1.1 [95% CI −7.8 to 9.4], *p =* 0.81). At 2 years, there were no statistically or clinically significant between-group differences in any of the outcome measures. All 3 complications resulting in secondary surgery occurred in the operative group. The lack of blinding in patient-reported outcome assessment is a limitation of the study. Our assessor physiotherapists were, however, blinded.

**Conclusions:**

This trial found no significant difference in clinical outcomes at 2 years between surgery and non-operative treatment in patients 60 years of age or older with displaced 2-part fractures of the proximal humerus. These results suggest that the current practice of performing surgery on the majority of displaced proximal 2-part fractures of the humerus in older adults may not be beneficial.

**Trial registration:**

ClinicalTrials.gov NCT01246167.

## Abstract 2

Antti Launonen and team assess surgery compared with locking plate and non-operative treatment for displaced two-part proximal humerus fractures in a superiority, assessor-blinded, randomized trial in six hospitals in Finland, Estonia, Sweden, and Denmark.

## Author summary

**Why was this study done?:**

- Proximal humerus fractures (PHFs) are among the most common fractures in older adults. The risks for suffering a PHF increase with age, especially after the age of 60 years.
- Non-operative treatment of non-displaced PHF is uniformly approved, but the treatment of displaced 2-part PHF remains controversial. Recently, several studies have reported a significant increase in the surgical treatment of 2-part PHF, mainly due to the introduction of locking plates suitable for treating osteoporotic bone.
- In this study, we investigated whether surgery with a locking plate is superior to non-operative treatment with a collar-cuff and early physiotherapy in the treatment of 2-part PHF involving the surgical neck.

**What did the researchers do and find?:**

- We randomly allocated 88 patients aged 60 years or older with displaced 2-part PHF into 2 groups. One group underwent surgery with a locking plate and the other conservative (non-operative) treatment.
- We recorded the Disabilities of Arm, Shoulder, and Hand (DASH) score, other measures of shoulder function and quality of life, and complications at 2-year follow-up.
- We found no significant or clinically important between-group differences in all outcomes.

**What do these finding mean?:**

- This trial provides no evidence that surgery is superior to non-operative treatment in the treatment of displaced 2-part PHF involving the surgical neck in older adults.
- These results suggest that the current practice of performing surgery on the majority of displaced 2-part proximal fractures of the humerus in older adults may not be beneficial.

## Introduction

Proximal humerus fractures (PHFs) are among the most common fractures in the older adult population [1,2]. In a Swedish nationwide study, the person-based incidence of PHF in adults was 175 per 100,000 person-years in women and 68 per 100,000 person-years in men [3]. The risk for having a PHF increases with age, especially after the age of 60 years [3,4].

The majority of PHFs are associated with a low-energy fall [4]. According to the published literature, minimally displaced or 2-part fractures constitute between 77% and 84% of all fractures [2,5]. Additionally, in another study, 76% are classified as AO Foundation/Orthopaedic Trauma Association (AO/OTA) A-class fractures [6], for which non-operative treatment can be considered. Fracture comminution and displacement, especially in 3- and 4-part fractures, on the other hand, are considered to be potential indications for operative treatment [7]. Recently, several studies have reported a substantial increase in surgery and claim it has now become current practice in the treatment of PHF, especially among the people aged over 60 years [3,8]. This increase has been mainly due to the introduction of locking plates, even though proper evidence of the superiority of surgery is lacking [8]. A few randomized controlled trials have compared operative treatment (plating or hemi-endoprosthesis) with non-operative treatment in displaced 3- and 4-part fractures [9–12]. The PROFHER trial, in which the majority of the fractures were displaced according to Neer criteria, also included 2-part fractures [12]. The study showed no differences between operative treatment and non-operative treatment based on a patient-reported outcome measure (PROM) or quality of life score. Based on the most recent Cochrane review as well as on other meta-analyses, no statistically or clinically significant difference in PROMs between operative and non-operative treatment has been observed [13–15].

It could be hypothesized that patients with displaced (defined by Neer criteria as more than 1 cm or 45 degrees) 2-part fractures [16] would benefit from operative treatment with a locking plate, but there is a scarcity of knowledge of the treatment outcomes associated with displaced 2-part fractures. We therefore conducted a multicenter, randomized, controlled efficacy trial that compared operative treatment with a locking plate with non-operative treatment in patients aged 60 years or over with 2-part displaced PHFs [17].

## Methods

### Study design

This PHF trial was a superiority, multicenter NITEP (Nordic Innovative Trial to Evaluate osteoPorotic fractures) group trial. The trial was conducted at 6 hospitals in 4 Northern European countries (Finland, Sweden, Denmark, and Estonia) [17]. All collaborating hospitals are emergency hospitals that routinely treat trauma patients. A specialized upper extremity team at each participating hospital provided the allocated treatment for the randomized patients, who were recruited between February 2011 and April 2016. The patients were informed about the treatment options during a pre-interviewing session, and all eligible patients gave their written informed consent before the randomization and initialization of the allocated treatment. The trial protocol was approved by the Regional Ethics Committee of Tampere University Hospital. In addition, the ethical committees of all participating hospital districts granted their ethical approval, and each hospital provided trial authorization before starting recruitment. Furthermore, independent steering and monitoring committees inspected the trial. The trial was designed by members of the protocol committee, and details of the trial design and methods have been published elsewhere [17].

In the protocol [17], 2 trial strata were described. Stratum I comprised 2-part fractures and stratum II comprised 3- and 4-part fractures. It was foreseen in the original protocol that stratum I would commence first, and therefore the reporting was planned separately for each protocol. At present, stratum II is still in the recruitment phase (159 of 218 patients recruited by 2 April 2019), which is scheduled to be completed during 2019. The analysis and reporting of these patients will start after the 2-year follow-up period. The trial protocol was signed by the principal investigator at each participating hospital in order to respect the approved treatment strategies.

The protocol was held in an Investigator Site File (ISF), and good clinical practice was the guiding principal in maintaining the study. The local investigators and the trial manager gathered the data. We used a prospective, randomized, open-label, blinded-endpoint design, where patients knew their allocation, but the persons who collected and analyzed the data and the authors were unaware of the study-group allocation of the patients. The authors vouch for the accuracy and completeness of the reported data and analyses, and the fidelity of the study to the protocol. The authors wrote the manuscript and made the decision to submit the manuscript for publication. This study is reported as per the Consolidated Standards of Reporting Trials (CONSORT) guideline (S1 CONSORT Checklist).

### Patients

Patients aged 60 years or over with displaced 2-part low-energy PHF (in which the fracture line emerges through the surgical or anatomical neck) occurring less than 2 weeks before allocation and treatment onset were eligible for inclusion. Displacement was defined by Neer classification (displacement more than 1 cm or 45 degrees, with bony contact) [16]. Pre-allocation radiograph along with computed tomography (CT) confirmed the classification. Furthermore, 2 upper extremity trauma surgeons reviewed each patient before randomization to confirm the classification. Detailed inclusion and exclusion criteria are presented in S1 Appendix.

### Randomization and blinding

Randomization was performed at the patient level. Eligible patients were randomly assigned in a 1:1 ratio to undergo operative treatment with a locking plate or non-operative treatment. After enrollment, patients underwent randomization by means of a telephone call from the treating surgeon to the coordinating center’s research nurse. Sealed envelopes were used, and the pre-trial randomization sequence was generated with 10 blocks according to center and stratified by age (60 to 70 years, more than 70 years) due to the association between age and the measured outcomes. The trial was semi-blinded, and the outcome assessors**—**trained physiotherapists who otherwise did not take part in the study—were unaware of which treatment group patients belonged to, and patients were encouraged not to reveal their treatment group. In addition, patients wore a T-shirt to cover any scars on their shoulder.

### Surgical and postoperative procedures

The treatments in the operative and non-operative groups were standardized [17]. Patients who received operative treatment with a Philos locking plate (Synthes, Solothurn, Switzerland) were operated on by an upper extremity trauma surgeon. The operating surgeon had a minimum of 5 years’ experience in shoulder trauma, and thus any learning curve problems in the treatment were avoided. After treatment, both groups received the same physiotherapy described in more detail in S2 Appendix. A collar-cuff or sling was worn for 3 weeks to reduce pain, and pendulum movements were initiated from the first day possible (non-operative patients immediately and surgery patients from first post-op day). Elbow, wrist, and fingers were mobilized, and the use of the injured upper extremity in daily activities was encouraged. After 3 weeks, active range-of-motion exercises were initiated under the supervision of a physiotherapist. Both groups were offered supervised physiotherapy rehabilitation sessions. At the follow-up visits, the patients were asked about the number of sessions received.

### Outcome measures

The primary outcome was Disabilities of the Arm, Shoulder, and Hand (DASH) score measured at 2-year follow-up. The DASH is a PROM with 36 questions on coping in different everyday-life tasks [18]. The scale ranges from 0 to 100, with a low number indicating better function. The minimal clinically important difference (MCID) on the DASH has been estimated to be 10 to 15 [19–21]. Secondary outcomes included Constant–Murley score (CS) [22], the visual analogue scale for pain (VAS; 0 to 100 mm) [23], the quality of life questionnaire 15D [24], the EuroQol Group’s 5-dimension self-reported questionnaire EQ-5D(-3L) (with normal values from Finland) [25], and the Oxford Shoulder Score (OSS) [26]. The CS has a known wide interobserver variation, and therefore we arranged pre-trial training for the investigators in order to harmonize the measurements. The follow-up questionnaires included a separate section on adverse events (AEs), defined as untoward medical occurrences that may or may not have a causal relationship with the treatment administered. AEs were classified as serious AEs (SAEs) if they necessitated hospitalization or prolonged inpatient hospital care, or if they were life-threatening or resulted in death. Complications were also recorded (infection, nerve damage, bleeding, mal- or non-union, hardware problems). The data were collected during the research visits at 3 months, 6 months, 12 months, and 24 months.

The baseline and follow-up data acquired from the patients were stored in paper portfolios that were analyzed after the last patient’s 2-year follow-up visit in April 2018. The portfolios included the patients’ baseline characteristics, patient questionnaires, validated questionnaires, and case report files that contained information about allocation group, crossovers (treatment another than the randomized allocation), AEs, and SAEs.

### Ethical approval

Primary approval was received from the Regional Ethics Committee of Tampere University Hospital, ETL-code R10127. Participation was voluntary. All data are anonymous.

### Statistical analysis

The trial was powered to detect a MCID in the DASH score of at least 10 points with a standard deviation (SD) of 15 (effect size *d* = 0.67) points. The initial planned sample size was 74 patients, which, at a 5% significance level, would provide 80% power to detect an effect size of 0.67 for the comparison of the 2 different treatment methods. Because the anticipated loss to follow-up was higher than the originally expected 10%, it was decided to raise the number of recruited patients from 81 to 88, resulting in 44 patients per group. This and other changes to protocol are summarized in S3 Appendix. The baseline characteristics were analyzed with descriptive statistics. Although we stated in the original trial protocol published in 2012 that subgroup analysis with respect to smoking, fracture type, and other diseases would be carried out, this was not done due to the rarity of these baseline characteristics in our sample. For the primary analysis, the primary and secondary outcomes at 2 years were compared between study groups with 95% confidence intervals. The principal analysis of the trial was performed after all participants had completed the 2-year follow-up. Trial analyses were conducted according to a prespecified statistical plan with the use of R software, version 3.5. All analyses were on an intention-to-treat basis and included all randomized patients in the groups to which they were randomized. Six patients were lost immediately after the allocation or were missing data from all the time points, and these patients were excluded from all the analyses. For the remaining patients, each outcome was treated as a time series in the individual patients, and the methods of linear interpolation and last observation carried forward were used to impute the missing data. A per protocol analysis was also conducted as a sensitivity analysis for the primary outcome. Continuous outcomes were compared using the Student *t* test. For each comparison, the homogeneity of variance was tested using Levene’s test meeting the assumption of the Student *t* test. Due to the skewness of the PROMs, we also used Mann–Whitney U test to compare the ranks between groups. All appropriate statistical tests were 2-sided.

Before revealing the randomization code, the writing committee (APL, VMM, MKL, and BOS) developed and recorded 2 interpretations of the results based on a blinded review of the primary outcome data (treatment A compared with treatment B). Thus, we aimed to decrease the possible researcher bias when interpreting the results. Only after the members of the writing committee had agreed that there would be no further changes in the interpretations, and unanimous consensus over the analysis reached, was the randomization code revealed, the correct interpretation chosen, and the manuscript finalized.

### Patient and public involvement

There was no patient involvement during the designing, recruiting, or conducting of the trial. However, after publication and dissemination of the results, patient organizations, health policy makers, and the general public will be informed of the important findings of the trial by means of congresses, social media, and general newsfeeds.

## Results

**Fig 1:**
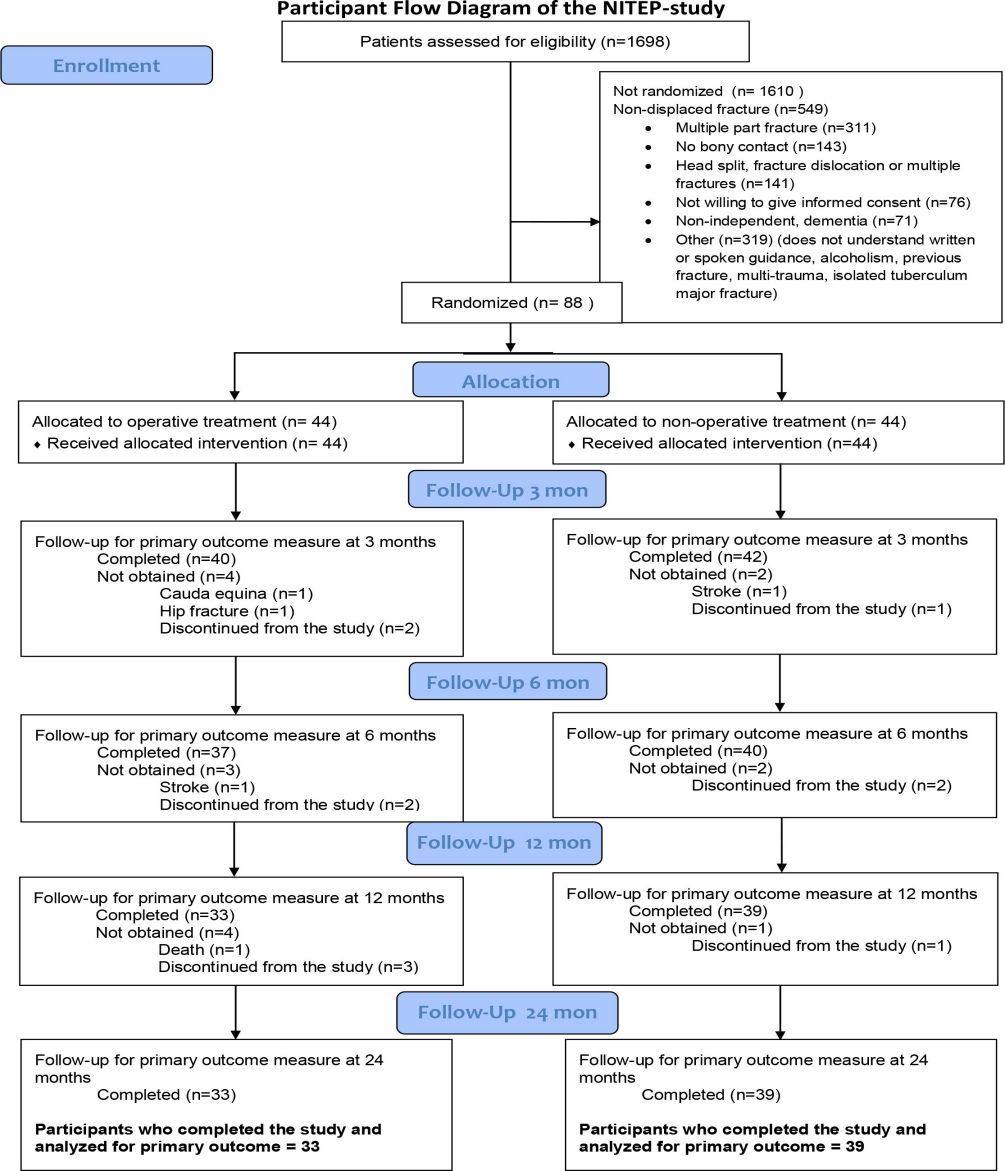
Participant flow diagram of the NITEP study.

Between February 2011 and April 2016, a total of 88 patients with PHF with surgical neck involvement were randomly assigned to undergo either operative treatment with a locking plate or non-operative treatment. In total, 44 patients were assigned to undergo operative treatment with a locking plate and 44 to non-operative treatment (Fig 1). The characteristics of the study population at the time of enrollment are shown in Table 1.

**Table 1:** Characteristics of 88 patients with a displaced 2-part proximal humerus fracture at baseline.

Patient characteristic	Surgical group *n =* 44	Non-operative group *n =* 44
Mean age (SD, range)	72 (7.4, 60–90)	73 (7.7, 60–86)
Age group		
60–69 years	17	20
70 years or above	27	24
Female sex	41 (95%)	39 (87%)
Fracture type: Surgical neck	44 (100%)	44 (100%)
Fracture on dominant side	15 (35%)	16 (36%)
Smoking	3	7
Diabetes	9	7
Neurological diseases	1	2
Mean DASH score (SD)*	17.1 (20.0)	15.9 (19.8)
Mean OSS (SD)*	41.7 (11.1)	40.5 (11.2)
Mean EQ-5D score (SD)*	0.87 (0.12)	0.85 (0.12)
Mean 15D score (SD)*	0.888 (0.087)	0.883 (0.090)

Data are number (percent) unless otherwise indicated.

*Baseline values measured before the fracture.

DASH, Disabilities of Arm, Shoulder, and Hand; OSS, Oxford Shoulder Score.

### Primary outcome measure

**Fig 2:**
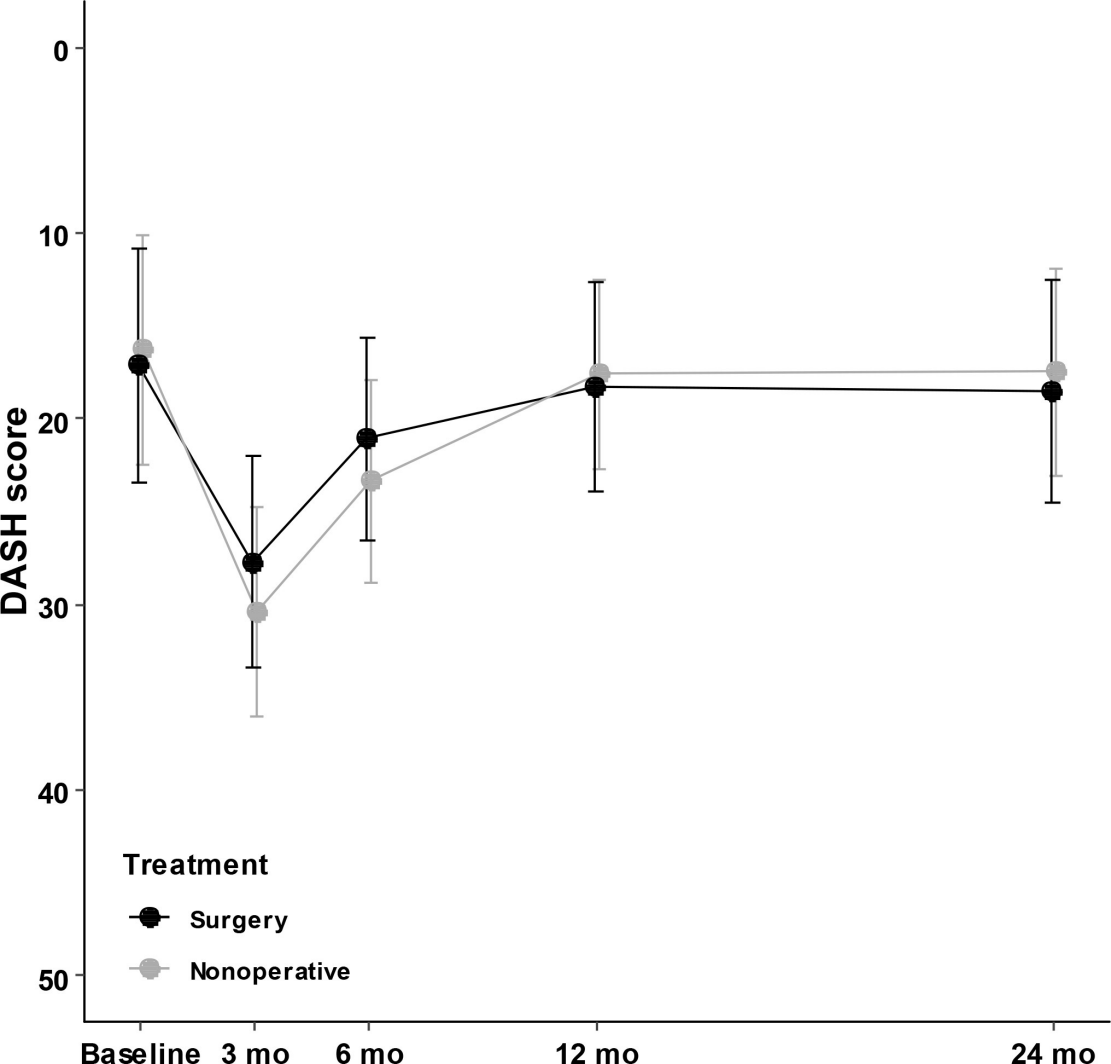
Between-group differences in mean DASH score (0 best, 100 worst) from baseline to 24 months. Vertical lines represent 95% confidence intervals. DASH, Disabilities of Arm, Shoulder, and Hand.

The mean DASH score at 2 years was 18.5 (SE 3.1) points for the operative treatment group and 17.4 (SE 2.8) points for the non-operative group. The between-group difference was 1.1 points (95% CI −7.8 to 9.4, *p =* 0.81). Detailed results are shown in Fig 2 and Table 2. At 2 years, there were no statistical or clinically significant between-group differences in the study groups. Sensitivity analysis using the available data showed similar results (S4 Appendix).

**Table 2:** Outcome measures for surgical and non-operative treatment groups at baseline, 3, 6, 12, and 24 months after displaced 2-part proximal humerus fracture.

Evaluation	Mean (SE)		Mean difference between the groups (95% CI)	*p*-Value of *t* test
	Surgical group	Non-operative group		
**DASH score**				
Baseline	17.1 (3.2)	15.9 (3.1)	1.2 (−8.1 to 9.8)	
3 months	27.7 (2.9)	30.5 (2.9)	−2.8 (−10.9 to 5.4)	0.51
6 months	21.1 (2.8)	23.4 (2.8)	−2.3 (−10.1 to 5.5)	0.57
12 months	18.3 (2.9)	17.6 (2.6)	0.7 (−7.1 to 8.4)	0.86
24 months	18.5 (3.1)	17.4 (2.8)	1.1 (−7.3 to 9.4)	0.81
**OSS**				
Baseline	41.7 (1.8)	40.5 (1.8)	1.2 (−3.8 to 6.2)	
3 months	32.6 (1.5)	33.0 (1.6)	−0.4 (−4.8 to 4.1)	0.87
6 months	39.3 (1.3)	38.2 (1.4)	1.1 (−2.7 to 4.9)	0.57
12 months	40.4 (1.4)	40.4 (1.3)	0 (−3.8 to 3.7)	0.98
24 months	40.2 (1.5)	41.5 (1.4)	−1.3 (−5.3 to 2.8)	0.54
**Constant–Murley score**				
6 months	57.3 (3.1)	55.1 (3.1)	2.2 (−5.7 to 10.1)	0.58
12 months	64.8 (3.1)	62.5 (3.4)	2.3 (−5.8 to 10.3)	0.58
24 months	68.0 (3.2)	66.0 (3.3)	2.0 (−5.6 to 9.6)	0.60
**EQ-5D**				
Baseline	0.87 (0.02)	0.85 (0.02)	0.02 (−0.04 to 0.07)	
3 months	0.84 (0.02)	0.82 (0.02)	0.02 (−0.03 to 0.07)	0.39
6 months	0.83 (0.02)	0.85 (0.02)	−0.02 (−0.07 to 0.03)	0.48
12 months	0.88 (0.02)	0.90 (0.02)	−0.02 (−0.08 to 0.02)	0.18
24 months	0.87 (0.02)	0.89 (0.02)	−0.02 (−0.08 to 0.02)	0.27
**VAS**				
Baseline	48.7 (4.8)	52.8 (4.9)	−4.1 (−17.7 to 9.5)	
3 months	22.2 (3.6)	21.7 (2.7)	0.5 (−9.0 to 9.9)	0.92
6 months	15.7 (3.3)	13.8 (2.4)	1.9 (−6.1 to 10.0)	0.63
12 months	13.0 (3.3)	13.0 (3.1)	0.0 (−9.0 to 8.9)	0.99
24 months	11.5 (3.3)	9.9 (2.7)	1.6 (−6.8 to 9.9)	0.72
**15D**				
Baseline	0.888 (0.014)	0.883 (0.015)	0.005 (−0.036 to 0.045)	
3 months	0.880 (0.015)	0.887 (0.014)	0.003 (−0.047 to 0.033)	0.84
6 months	0.894 (0.015)	0.890 (0.014)	0.004 (−0.037 to 0.046)	0.91
12 months	0.884 (0.020)	0.887 (0.016)	−0.003 (−0.053 to 0.047)	0.60
24 months	0.862 (0.029)	0.879 (0.017)	−0.016 (−0.084 to 0.049)	0.60

Baseline values measured before fracture with the exception of VAS, which was recorded after the fracture.

DASH, Disabilities of Arm, Shoulder, and Hand; OSS, Oxford Shoulder Score; VAS, visual analogue scale for pain.

When stratified by age, the between-group difference in mean DASH score (with *t* test) at 2 years in patients aged 60–70 years was −0.2 (95% CI −10.1 to 9.7, *p =* 0.97). The between-group difference in mean DASH score at 2 years in patients aged more than 70 years was 1.7 (95 CI −11.2 to 14.5, *p =* 0.79). An additional secondary sensitivity analysis, requested by an external reviewer, showed no significant between-group difference in the number of patients with a DASH score difference of 10 points or more between baseline and 24 months (*p =* 0.30); see S4 Appendix.

### Secondary outcomes

At 2 years, we found no statistical or clinically significant between-group differences in study groups for the CS (mean 68.0 points [SE 3.2] in the operative treatment group compared with 66.0 points [SE 3.3] in the non-operative group; between-group difference 2.0 [95% CI −5.6 to 9.6], *p =* 0.6). For the OSS, the mean was 40.2 points (SE 1.5) in the operative treatment group compared with 41.5 points (SE 1.4) in the non-operative group (between-group difference −1.3 [95% CI −5.3 to 2.8], *p =* 0.54). For the EQ-5D, the mean was 0.87 points (SE 0.02) in the operative treatment groups compared with 0.89 points (SE 0.02) in the non-operative group (between-group difference −0.02 [95% CI −0.08 to 0.02], *p =* 0.27). For the VAS, the mean was 11.5 points (SE 3.3) in the operative treatment group compared with 9.9 points (SE 2.7) in the non-operative group (between-group difference 1.6 [95% CI −6.8 to 9.9], *p =* 0.72). For the 15D, the mean was 0.862 points (SE 0.029) in the operative treatment groups compared with 0.879 points (SE 0.017) in the non-operative group (between-group difference −0.016 [95% CI −0.084 to 0.049], *p =* 0.6). For physiotherapy, the median number of visits was 4 in the operative group (range 0 to 25 visits) compared with 4 in the non-operative group (range 0 to 28 visits) (*p =* 0.38).

### Complications

At 2 years, there were 3 complications. They all occurred in the operative group and required subsequent surgery. No other complications were detected. One patient fell and sustained a peri-implant fracture distal to the tip of the plate. This fracture was treated with open reduction and internal fixation by long anatomical locking plate. Two patients had implant failures because proximal screws migrated into the joint. These patients were treated with revision operation, and the screws were changed. The mean time to revision operation was 5.5 months (range 1.3 to 12.4 months). In the non-operative group, there were no complications; however, the difference between the groups was not statistically significant (3 versus 0, *p =* 0.24). One patient died during the study period. The death was not related to the trial. No patient withdrew from the study because of adverse effects.

## Discussion

The present investigation is, to our knowledge, the first prospective randomized controlled trial to compare non-operative and operative treatment with Philos plate in patients aged 60 years or older with displaced 2-part PHFs. This trial provides no evidence that surgery is superior to non-operative treatment in 2-year follow-up. Furthermore, we found no clinically or statistically significant between-group differences in any of the outcomes measured, including DASH (our primary outcome measure), CS, OSS, EQ-5D, 15D, VAS, complications, and mortality.

The DASH score, OSS, and CS are outcome measures commonly used to assess upper limb function after PHF. We acknowledge the limits of the DASH score as being a patient-reported scoring tool to assess symptoms and physical disability in the arm. However, it is also widely used in PHFs, it has a known MCID [19] and known average values for the general population, and it is validated in all Nordic countries.

The mean DASH scores in both of groups improved between 3 months and 6 months and between 6 months and 1 year. However, the scores did not improve after 1 year, when they reached the levels of baseline scores. The baseline values in our study are in line with the average values calculated from the general population, which constitutes both healthy individuals and people with disabilities of the upper limb [27]. Based on our results, we can conclude that the final function and satisfaction, measured by DASH, is obtained 1 year after the trauma.

We are not aware of any other published randomized trial that makes this specific comparison for displaced 2-part fractures of the surgical neck of the proximal humerus in older adults only. These are the most common displaced PHFs [5,28]. The PROFHER trial, which also included younger patients, is the only other trial to our knowledge that included these fractures. Although the study design, study settings, and age criteria differ between our study and PROFHER, both trials found no significant differences between surgical and non-operative treatment.

Although the difference in complications leading to revision operation in the present study did not reach statistical significance, the operative group had several complications, whereas the non-operative treatment group had none of any kind. All 3 complications leading to revision operation occurred within the first year (mean 5.5 months) after the surgery. In contrast, in the trial by Olerud et al., less than a quarter (7% from a total of 30%) of re-operations took place in the first year of follow-up [10]. In the PROFHER trial, 70% of secondary surgeries occurred within the first year [12].

One strength of the study lies in the fact that the trial participants had sustained injuries that are typically considered for surgical intervention. Second, both interventions and treatment protocols were representative of good practice, and this included the fact that all of the operations were performed by consultant surgeons. Additionally, the Philos locking plate used in this study is the most commonly used implant in current practice in Northern European countries as well as in many other countries. Moreover, only patients with 2-part PHFs involving the surgical neck were included in the trial. Previously, there has been disagreement over recognizing different fracture categories [29]. In our previous publication, however, we found substantial intra- and interobserver agreement in re-categorized Neer classification [30]. Finally, the dropout rate in our trial was 18%, which, according to Furlan et al., is acceptable, especially among patients with high mean age and numerous comorbidities [31].

The main weaknesses of the study were that patients were not blinded to the treatment, although outcome assessor physiotherapists were. Further, even though a priori power analysis was performed in order to establish the sample size required to adequately differentiate a true lack of clinically meaningful difference, there is the possibility the study was underpowered. Our main outcome variable, the DASH questionnaire, focuses on functional status and symptoms in general rather than on one specific anatomical region or specific disease entity. There is, however, no validated outcome measure specifically for shoulder fractures. In addition, as in the general PHF population, most patients in this study were women. Therefore, the results are representative and reliable for the female population.

The study group comprised patients with 2-part fracture of the proximal humerus with contact among the fragments. Hence, the results of this study are not applicable to fractures with no contact, head-split fractures, or dislocated fractures. For a better overview of patients included in the trial, we have uploaded anonymous plain radiographs to the NITEP group website along with 2-year DASH-values (http://www.nitep.eu/rtg).

### Conclusions

The results of this superiority, semi-blinded randomized controlled trial show that surgery is not more beneficial than non-operative treatment in patients 60 years of age or older with displaced 2-part PHF. These results suggest that the current practice of performing surgery on the majority of displaced 2-part proximal fractures of the humerus in older adults may not be beneficial. Mean DASH scores already returned to the baseline level at 1 year, and therefore 1-year follow-up will be sufficient for future PHF trials with non-operative treatment and plating.

## Supporting information

S1 Consort Checklist (DOC)

S1 Appendix Inclusion and exclusion criteria of the NITEP study. (DOCX)

S2 Appendix Description of post-treatment physiotherapy. (DOCX)

S3 Appendix Description of changes to the published protocol. (DOCX)

S4 Appendix New additional analyses. (DOCX)

## Abbreviations

AE: adverse event
CS: Constant–Murley score
DASH: Disabilities of Arm, Shoulder, and Hand
MCID: minimal clinically important difference
OSS: Oxford Shoulder Score
PHF: proximal humerus fracture
PROM: patient-reported outcome measure
SAE: serious adverse event
VAS: visual analogue scale for pain
