# Supplementary material for: Operative versus non-operative treatment for 2-part proximal humerus fracture: A multicenter randomized controlled trial
Source: PLoS Med. 2019 Jul 18;16(7):e1002855. doi: 10.1371/journal.pmed.1002855 (PMC6638737; doi:10.1371/journal.pmed.1002855)
Supplement: S1 Appendix — (DOCX) [file pmed.1002855.s002.docx]

**APPENDIX 1**

**Inclusion and exclusion criteria for the NITEP-study**

***Inclusion criteria***

- Low energy proximal humerus displaced (displacement more than 1 cm or 45 degrees) two-part fracture in which the fracture line emerges through the surgical (or anatomic) neck

***Exclusion criteria***

- Refusal to participate in the study
- Under 60 years of age
- Not independent
- Dementia and/or institutionalized
- Does not understand written and spoken guidance in either Finnish or Swedish
- Pathologic fracture or a previous fracture of the same proximal humerus
- Alcoholism or drug addiction, e.g., in the emergency department, breathalyzer indicates blood alcohol concentration of more than 2‰
- Other injury to the same upper limb requiring surgery
- Major nerve injury (e.g., complete radial- or axillary nerve palsy)
- Rotator cuff tear arthropathy
- Open fracture
- Multi-trauma or -fractured patient
- Fracture dislocation or head-splitting fracture
- Non-displaced fracture
- Isolated fracture of the major or minor tubercle
- Gross displacement of the fracture fragments (no bony contact between fracture parts or the humerus shaft is in contact with the articular surface)
- Any medical condition that excludes surgical treatment
