# Supplementary material for: Operative versus non-operative treatment for 2-part proximal humerus fracture: A multicenter randomized controlled trial
Source: PLoS Med. 2019 Jul 18;16(7):e1002855. doi: 10.1371/journal.pmed.1002855 (PMC6638737; doi:10.1371/journal.pmed.1002855)
Supplement: S2 Appendix — (DOCX) [file pmed.1002855.s003.docx]

Appendix 2

The following post-operative physiotherapy was carried out:

Patients were instructed with regard to joint mobilization by a physiotherapist during hospitalization. Patients received a written aftercare protocol with detailed pictures. A collar-cuff or a sling was used for three weeks to relieve pain. During the first three weeks, pendulum exercises were allowed, and free joint mobilization and normal limb activation throughout the treatment was strongly supported.

Active range-of-motion exercises, as permitted by pain, begun at three weeks. Physiotherapist contacts were arranged for three and six weeks after the beginning of the treatment, and all patients were scheduled to have five physiotherapist contacts within the first three months. For the physiotherapy, the median number of visits was 4 in the operative group (range 0 to 25 visits) compared with 4 in the non-operative group (range 0 to 28 visits) (p=0.38).
