# Supplementary material for: Operative versus non-operative treatment for 2-part proximal humerus fracture: A multicenter randomized controlled trial
Source: PLoS Med. 2019 Jul 18;16(7):e1002855. doi: 10.1371/journal.pmed.1002855 (PMC6638737; doi:10.1371/journal.pmed.1002855)
Supplement: S3 Appendix — (DOCX) [file pmed.1002855.s004.docx]

Appendix 3 - Changes from protocol

A few changes occurred to the original protocol[1] during the trial. These changes and clarifications are explained below.

**Notes of the Stratum II**

As clarified in the text, this trial included to two strati. Stratum I, reported here, comprised two-part fractures and stratum II comprised three- and four-part fractures. It was foreseen in the original protocol that stratum I would commence first, and therefore the reporting was planned separately for each protocol. At present, stratum II is still in the recruitment phase that is scheduled to be completed during 2019. The analysis and reporting of these patients will start after the two-year follow-up period.

**Sample size calculation: A corrective note**

When assuming an effect size of a 10-point difference in the DASH score and a standard deviation of 15 points, the estimated sample size in each group was 37 patients (delta=10, SD=15, α=0.05 and power=0.08). Therefore, the original sample size was 74 patients. In the original sample size calculation, 10% was used as a dropout rate, resulting in 81 patients. However, at a site-manager’s meeting (13^th^ of January 2016 in Stockholm) the drop-out rate was reported to be higher, due to multiple reasons, but mostly patient withdrawal because of old age, unwillingness to join follow-up visits and difficulties with transportation. Therefore, the drop-out rate was recalculated to be 20%. With a drop-out rate of 20%, the re-calculated new sample size became 88 patients, which is the figure reported in this article.

**Used Hardware in the trial**

In the protocol, we stated the use of Philos locking (Synthes®) plate in stratum I and stratum II. Here we confirm that operative treatment was carried out with the Philos plate for all patients of stratum I.

**Rehabilitation protocol**

Patients used collar-cuff or a sling to relieve the pain. The physiotherapy received is described in Appendix II. Most trial participants recorded attending fewer than the 5 scheduled physiotherapy sessions; however, the median number of visits was 4 in both groups.

**Subgroup and secondary analyses**

In the protocol we state the following: “In the subgroup analysis, the effect of age, sex, fracture group, smoking and other diseases will be evaluated against the ROM, OSS, Constant-Murley, and overall quality of life after fracture”.

However, we did not carry out these planned sub-group analyses because the prevalence of these variables was too low.

As requested at peer-review, we conducted a secondary analysis to determine how many in each group had a poor outcome (DASH difference 10 or more points from baseline); see Appendix IV.

1. Launonen AP, Lepola V, Flinkkila T, Strandberg N, Ojanpera J, Rissanen P, et al. Conservative treatment, plate fixation, or prosthesis for proximal humeral fracture. A prospective randomized study. BMC Musculoskelet Disord. 2012;13:167. Epub 2012/09/08. doi: 10.1186/1471-2474-13-167. PubMed PMID: 22954329; PubMed Central PMCID: PMC3520878.
