# Supplementary material for: Operative versus non-operative treatment for 2-part proximal humerus fracture: A multicenter randomized controlled trial
Source: PLoS Med. 2019 Jul 18;16(7):e1002855. doi: 10.1371/journal.pmed.1002855 (PMC6638737; doi:10.1371/journal.pmed.1002855)
Supplement: S4 Appendix — (DOCX) [file pmed.1002855.s005.docx]

Appendix 4 – New additional analyses

Sensitivity analysis: DASH results using available data only

When calculating results with existing data only (without imputation) the results were as follows:

|  | *N surgical group* | *N non-surgical group* | *Mean (SD) surgical group* | *Mean (SD) non-surgical group* | *Mean difference (95% CI)* |
| --- | --- | --- | --- | --- | --- |
| *DASH 3 months* | *36* | *38* | *27.1 (19.1)* | *31.9 (18.7)* | *-4.8 (95% CI -13.6 to 3.9)* |
| *DASH 6 months* | *35* | *38* | *21.0 (17.4)* | *23.4 (18.0)* | *-2.4(95% CI: -10.5 to 5.7)* |
| *DASH 12 months* | *35* | *39* | *18.0 (18.5)* | *17.5 (17.0)* | *0.5(95% CI: -7.6 to 8.6)* |
| *DASH 24 months* | *33* | *39* | *17.4 (19.7)* | *17.4 (18.7)* | *0(95% CI -8.8 to 8.8)* |

Sensitivity analysis based on baseline values

Baseline DASH adjusted primary outcome (i.e., DASH 24 months) was done using linear regression treatment group and baseline as independent variables. Regression coefficient for treatment group indicating the mean difference between groups was -0.1 (95% -8.3 to 8.0).

New secondary analysis
As requested at peer-review, we conducted a secondary analysis to determine how many in each group had a poor outcome, defined as a DASH difference 10 or more points from baseline. We found 7 patients in the operative group and 12 in the non-operative group had DASH score difference 10 points or more between baseline and 24 months (p=0.30).
